# HIV-1 subtype C predicted co-receptor tropism in Africa: an individual sequence level meta-analysis

**DOI:** 10.1186/s12981-020-0263-x

**Published:** 2020-02-07

**Authors:** Nontokozo D. Matume, Denis M. Tebit, Pascal O. Bessong

**Affiliations:** 1 HIV/AIDS & Global Health Research Programme, and Department of Microbiology, University of Venda, Thohoyandou, 0950 South Africa; 2 Global Biomed Scientific LLC, P.O. Box 2368, Forest, VA 24551 USA

**Keywords:** HIV-1 subtype C, Early infections, Chronic infections, Co-receptor tropism, Africa

## Abstract

**Background:**

Entry inhibitors, such as Maraviroc, hold promise as components of HIV treatment and/or pre-exposure prophylaxis in Africa. Maraviroc inhibits the interaction between HIV Envelope gp120 V3-loop and CCR5 coreceptor. HIV-1 subtype C (HIV-1-C) is predominant in Southern Africa and preferably uses CCR5 co-receptor. Therefore, a significant proportion of HIV-1-C CXCR4 utilizing viruses (X4) may compromise the effectiveness of Maraviroc. This analysis examined coreceptor preferences in early and chronic HIV-1-C infections across Africa.

**Methods:**

African HIV-1-C Envelope gp120 V3-loop sequences sampled from 1988 to 2014 were retrieved from Los Alamos HIV Sequence Database. Sequences from early infections (< 186 days post infection) and chronic infections (> 186 days post infection) were analysed for predicted co-receptor preferences using Geno2Pheno [Coreceptor] 10% FPR, Phenoseq-C, and PSSMsinsi web tools. V3-loop diversity was determined, and viral subtype was confirmed by phylogenetic analysis. National treatment guidelines across Africa were reviewed for Maraviroc recommendation.

**Results:**

Sequences from early (n = 6316) and chronic (n = 7338) HIV-1-C infected individuals from 10 and 15 African countries respectively were available for analyses. Overall, 518/6316 (8.2%; 95% CI 0.7–9.3) of early sequences were X4, with Ethiopia and Malawi having more than 10% each. For chronic infections, 8.3% (95% CI 2.4–16.2) sequences were X4 viruses, with Ethiopia, Tanzania, and Zimbabwe having more than 10% each. For sequences from early chronic infections (< 1 year post infection), the prevalence of X4 viruses was 8.5% (95% CI 2.6–11.2). In late chronic infections (≥ 5 years post infection), X4 viruses were observed in 36% (95% CI − 16.3 to 49.9), with two countries having relatively high X4 viruses: South Africa (43%) and Malawi (24%). The V3-loop amino acid sequence were more variable in X4 viruses in chronic infections compared to acute infections, with South Africa, Ethiopia and Zimbabwe showing the highest levels of V3-loop diversity. All sequences were phylogenetically confirmed as HIV-1-C and clustered according to their co-receptor tropism. In Africa, Maraviroc is registered only in South Africa and Uganda.

**Conclusions:**

Our analyses illustrate that X4 viruses are present in significantly similar proportions in early and early chronic HIV-1 subtype C infected individuals across Africa. In contrast, in late chronic infections, X4 viruses increase 3–5 folds. We can draw two inferences from our observations: (1) to enhance the utility of Maraviroc in chronic HIV subtype C infections in Africa, prior virus co-receptor determination is needed; (2) on the flip side, research on the efficacy of CXCR4 antagonists for HIV-1-C infections is encouraged. Currently, the use of Maraviroc is very limited in Africa.

## Background

Data from the Joint United Nations Programme on HIV/AIDS (UNIAIDS) shows that about 38 million people were living with HIV infection at the end of 2018, 68% of this number are in Africa [1]. The UNAIDS 90–90–90 target translates to 90% of all persons to be tested for HIV, 90% of those infected should be on treatment, and 90% of those on treatment should have their viral load suppressed to undetectable levels. In this scheme, treatment is expected to act as a prevention tool, since the chances of transmission is highly reduced when viral load is undetectable [2–5]. Combination antiretroviral therapy (cART) is the gold standard for the management of HIV infections. In most developing countries, nucleoside and non-nucleoside reverse transcriptase inhibitors are the backbone of all first and second line of antiretroviral regimens in adults, with a boosted protease component for children in first line treatment [6–9].

The goal of cART is to rapidly reduce HIV viral load to undetectable levels, thereby permitting the reconstitution of immune function as measured by rising levels of CD4+ cell counts. The increasing ease of access to cART across Africa has tremendously reduced morbidity and mortality due to HIV infection [10–14]. However, treatment is not curative, and a significant proportion of individuals will fail first line and second line regimens making them legible for salvage therapy. Maraviroc, an entry inhibitor, is gaining significance as part of treatment regimens in the United States and elsewhere [15–17], but there is little documentation of its use in Africa, even as salvage therapy.

Maraviroc inhibits viral entry by prohibiting the interaction between HIV Envelope gp120 V3-loop and the CCR5 co-receptor, following the interaction of Gp120 with the CD4 molecule [18–21]. High tolerability, safety, and efficacy in viral reduction in both treatment experienced and naïve patients have been demonstrated for Maraviroc, making it a valuable treatment option against HIV/AIDS [22]. It has been reported that amino acid substitutions, particularly by glycine, in the V3 loop crown motive may reduce the binding efficiency of gp120 to CCR5 [23]. Globally, about 47% of infections are due to HIV-1 subtype C (HIV-1-C) [24], and HIV-1-C overwhelmingly dominates infections in Southern Africa [25–27]. It is not conclusive how and why HIV-1-C appears to be more transmissible than other members of HIV Group M and how it became the dominant variant in Southern Africa. However, some Ex-vivo pathogenic fitness studies and long-term natural history cohorts in Uganda and Zimbabwe have suggested that subtype C is the least fit subtype among HIV-1 group M. Their lower virulence leads to longer asymptomatic periods which could explain their continuous dominance and expansion in the HIV global pandemic [28, 29]. Further, phylogeography reconstruction models of HIV polymerase sequences [30], have shown that HIV-1-C may have originated from Lubumbashi and Mbuji-Mayi (cities in the Democratic Republic of Congo) from where it was transmitted to Southern Africa, facilitated by the developed and busy road and rail networks in the 1960s. In addition [31], had demonstrated that conserved V1–V2 loops and V3-316T, which occur at higher frequencies in HIV-1-C, increase viral infectivity; and proposed that this could be responsible for the relatively high transmissibility of HIV-1-C heterosexually.

A significant majority of the initial infecting HIV-1-C viruses utilize CCR5. However, the presence of a significant proportion of CXCR4 utilizing viruses (X4) in chronic HIV-1-C infection [32–34] might compromise the effectiveness of CCR5 antagonists, such as Maraviroc, when included as components in salvage therapy. In the current analyses, co-receptor preference in early and chronic HIV-1-C infections across Africa was examined, using sequences from the Los Alamos HIV Sequence Database, with the view of understanding the co-receptor preference landscape of the epidemiologically important HIV-1-C across the continent. We also examine literature for indications of active use of Maraviroc in African countries. The association of viral tropism to stage of infection and mode of transmission were examined. We observed that X4 viruses are present in similar proportions in early (less than 6 months post infection) and early chronic (less than 1 year post infection) HIV-1 subtype C infected individuals across Africa. On the contrary, in late chronic infections, there is a significant 3–5 fold increase in X4 viruses. Although there is currently a limited use of Maraviroc across Africa, our findings could be useful in the development of treatment management guidelines in regions where HIV-1 subtype C drives the epidemic.

## Methods

### Sequence search and extraction

HIV-1 subtype C Gp120 V3-loop Sanger generated sequences were extracted from the Los Alamos HIV Sequence Database (https://www.hiv.lanl.gov/content/index) using the sequence search interface. Firstly, sequences were searched and extracted for each African country based on early and chronic infections. In this study, early infection was defined as the period comprising HIV infection, seroconversion, and recent infection [35] and chronic infection was defined as the period post recent infection. Most of the sequences retrieved were generated when tests to measure HIV RNA, and thus detect acute infections were not available in many African countries. Secondly, extracted sequences were categorized according to the following: route of infection (mother-to-child transmission (MTCT) and heterosexual transmission), and disease progression (slow and rapid progressors). Problematic sequences and those with no data on seroconversion dates were excluded from the analyses.

### Co-receptor prediction and sequence analyses

**Fig. 1:**
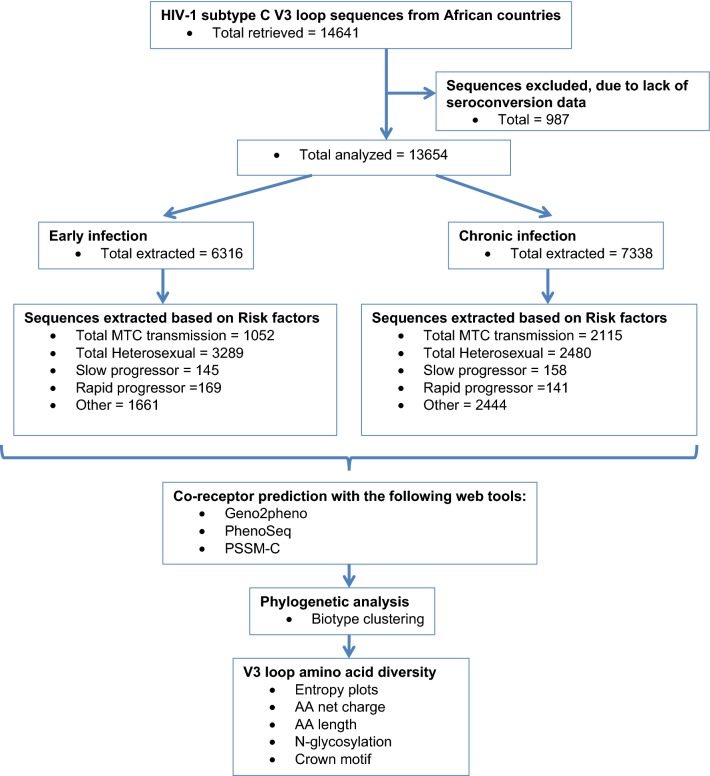
Flow chart illustrating the study procedure: sequence extraction, categorization, co-receptor prediction and diversity analysis

Sequences were classified as early (< 186 days post infection) and chronic infections (> 186 days post infection). Sub-classifications included early chronic infection (186 days to 1 year post infection) and late chronic infections (> 5 years post infection). Sequences were separately analysed for predicted co-receptor preferences using Geno2Pheno [Coreceptor] 10% FPR (https://coreceptor.geno2pheno.org/), Phenoseq-C (http://tools.burnet.edu.au/phenoseq/), and PSSMsinsi (https://indra.mullins.microbiol.washington.edu/webpssm/) web tools. Phenoseq-C and PSSMsinsi are particularly HIV-1-C based co-receptor prediction tools [36–39]. An inferred concordance of all three tools was used to assign the co-receptor biotype. Gp120 V3-loop diversity was determined for both R5 and X4 viruses by entropy plotting, amino acid length, net charge, N-glycosylation and crown motif examination. The genetic subtype was confirmed by phylogenetic analysis. Figure 1 illustrates an outline of the procedures used in the study.

### Active use of Maraviroc in Africa

To estimate the active use of Maraviroc in Africa, national ART guidelines and documents on ARV approvals were retrieved by Google search and reviewed for the recommendation and approval of Maraviroc. Search terms included: national ART guidelines AND [African country], and this was done for all African countries. We also reviewed the recent summary statement of HIV treatment regimens in Africa, 2017 [40].

### Statistical analyses

To statistically assess the differences between frequencies, a Fisher’s exact test was conducted; confidence intervals at the 95% threshold were calculated to estimate the interval estimate of a population mean; using an online GraphPad QuickCalcs tool (https://www.graphpad.com/quickcalcs/contingency1.cfm). Significant differences were implied when p-values were < 0.05.

## Results

HIV-1 subtype C Gp120 V3-loop co-receptor was predicted, analysed and categorized according to early or chronic infections in general, route of transmission (mother-to-child and heterosexual), and pathogenesis (slow and rapid progressors). In addition, the V3-loop amino acid diversity in terms of amino acid length, entropy, net charge and N-glycosylation sites were further analysed (Fig. 1). Sequences used for these analyses were obtained from the Los Alamos HIV Sequence database, spanned from 1998 through 2014 and originated from 15 out of 54 (27.8%) African countries. These countries were mostly from East and Southern Africa where subtype C is most prevalent. A total of 14,641 HIV-1-C gp120 V3-loop sequences were retrieved. Of these, 987 were excluded due to lack of seroconversion date, affording 13,654 sequences, of which 6316 were from early infections, and 7338 were from chronic infections (Table 1 and Fig. 1).

**Table 1:** Co-receptor prediction using sequences from African HIV-1 subtype C early (< 6 months of seroconversion) and chronically (> 6 months of seroconversion) infected individuals

African countries	Predicted biotype					
	Early			Chronic		
	Total (%)	X4 (%)	R5 (%)	Total (%)	X4 (%)	R5 (%)
Total sequences analyzed	6316	518 (8.2%; 95% CI 0.7–9.3)	5798 (91.8%; 95% CI 90.7–99.3)	7338	612 (8.34%; 95% CI 2.4–16.2)	6726 (91.7%; 95% CI 84.8–97.5)
Botswana (BW)	1401 (22.2)	68 (4.9)	1333 (95.1)	1604 (21.9)	96 (6)	1508 (94)
Burundi (BI)	NA	NA	NA	4 (0.05)	0	4 (100)
Congo (CD)	NA	NA	NA	18 (0.25)	0	18 (100)
Ethiopia (ET)	20 (0.32)	3 (15)	17 (85)	164 (2.2)	56 (34)	108 (66)
Gabon (GA)	NA	NA	NA	2 (0.03)	0	2 (100)
Gambia (GM)	NA	NA	NA	3 (0.04)	1 (33.3)	2 (66.6)
Guinea-Bissau (GW)	NA	NA	NA	7 (0.1)	0	7 (100)
Kenya (KE)	2 (0.03)	0	2 (100)	12 (0.2)	0	12 (100)
Malawi (MW)	2100 (33.2)	339 (16.1)	1761 (83.9)	2591 (35.3)	164 (6.3)	2427 (93.7)
Rwanda (RW)	109 (3)	0	109 (100)	8 (0.1)	0	8 (100)
Senegal (SN)	3 (0.06)	0	3 (100)	NA	NA	NA
South Africa (ZA)	1246 (19.7)	59 (4.7)	1187 (95.3)	1151 (15.7)	121 (10.5)	1030 (89.5)
Tanzania (TZ)	86 (1.4)	5 (5.8)	81 (94.2)	376 (5.1)	62 (16.5)	314 (83.5)
Zambia (ZM)	1345 (21.3)	44 (3.3)	1301 (96.7)	1353 (18.4)	100 (7.4)	1253 (92.6)
Zimbabwe (ZW)	4 (0.06)	0	4 (100)	44 (0.6)	12 (26)	32 (74)
Uganda (UG)	NA	NA	NA	1 (0.01)	0	1 (100)

*NA* not available

### Co-receptor biotype prediction

A total of 6316 HIV Gp120 V3-loop Sanger generated sequences from early HIV-1 subtype C infections were available from 10 countries, namely; Botswana, Ethiopia, Kenya, Malawi, Rwanda, Senegal, South Africa, Tanzania, Zambia, and Zimbabwe. For chronic infections, 7338 sequences were available from 15 countries, namely; Botswana, Burundi, DR Congo, Ethiopia, Gabon, Gambia, Guinea Bissau, Kenya, Malawi, Rwanda, South Africa, Tanzania, Uganda, Zambia, and Zimbabwe. Analyses of all early infection sequences showed that 518/6316 (8.2%; 95% CI 0.7–9.3) were of X4 variant while 5798 (91.8%; 95% CI 90.7–99.3) were R5 (Table 1). Ethiopia (3/20; 15.0%) and Malawi (339/2100; 16.1%) had more than 10% of X4 using viruses. For all chronic infections, 612/7338 (8.34%; 95% CI 2.4–16.2) of the sequences were X4-tropic, with four countries, Ethiopia, South Africa, Tanzania, and Zimbabwe each having about 10% or more (Table 1). Overall, there was no difference in the proportion of X4 viruses in early (8.2%) versus all chronic infections (8.3%) (p = 0.8; X^2^ = 0.064). When sequences, from early chronic infections (> 186 days to < 1 year post-infection) were considered, the prevalence of X4 viruses was 156/1832 (8.5%; 95% CI 2.6–11.2). For chronic infections ≥ 186 day, X4 viruses were present in 64/179 (36%; 95% CI − 16.3 to 49.9) of the study population (p = 0.0001; X^2^ = 65.257), with two countries having relatively high X4 prevalence: South Africa (43%; 58/136) and Malawi (24%, 6/25).

### Co-receptor biotype prediction according to mode of transmission

Of the 6316 acute HIV infection sequences, 1052 were from cases of MTCT. Of these, only 46/1052 (4.4%; 95% CI − 1.5 to 6.5) were X4-tropic. Country-wise, Malawi and Tanzania marginally had more X4 viruses (6% and 6.1% respectively) in early infections (Table 2). In comparison, among chronic infections, 143/2115 (6.8%; 95% CI − 10.9 to 31.5) were X4-tropic viruses, with South Africa having a significantly higher number of X4 viruses (40.4%; p = 0.001; X^2^ = 30.288). The proportion of X4 viruses in early MTCT sequences was not significantly different from chronic MTCT cases; 4.4% (46/1052) versus 6.7% (143/2115) (p = 0.535; X^2^ = 0.385) respectively (Table 2).

**Table 2:** Co-receptor prediction using sequences from HIV-1 subtype C mother-to-child and heterosexual transmissions

	Predicted biotype					
	Early			Chronic		
	Total (%)	X4 (%)	R5 (%)	Total (%)	X4 (%)	R5 (%)
Mother-to-child transmission						
Total sequence	1052	46 (4.4%; 95% CI − 1.5 to 6.5)	1006 (95.6%; 95% CI 93.5, 101.5)	2115	143 (6.8%; 95% CI − 10.9 to 31.5)	1972 (93.2%; 95% CI 68.5–110.9)
Malawi (MW)	691 (65.6)	41 (5.9)	650 (94.1)	1693 (80)	63 (3.7)	1630 (96.3)
South Africa (ZA)	11 (1.1)	0	11 (100)	151 (7.1)	61 (40.4)	90 (59.6)
Tanzania (TZ)	49 (4.7)	3 (6.1)	46 (93.9)	1 (0.04)	0	1 (100)
Zambia (ZM)	297 (28.2)	2 (0.7)	295 (99.3)	263 (12.4)	19 (7.2)	244 (92.8)
Zimbabwe (ZW)	4 (0.4)	0	4 (100)	7 (0.33)	0	7 (100)
Heterosexual transmission						
Total sequences analyzed	3289	385 (11.7%; 95% CI − 1.8 to 13.9)	2904 (88.3%; 95% CI 86.4–101.8)	2480	231 (9.3%; 95% CI − 0.9 to 13.5)	2249 (90.7%; 95% CI 86.5–100.9)
Botswana (BW)	29 (0.9)	1 (3.4)	28 (96.6)	4 (0.16)	0	4 (100)
Ethiopia (ET)	1 (0.03)	0	1 (100)	10 (0.4)	0	10 (100)
Kenya (KE)	1 (0.03)	0	1 (100)	22 (0.7)	0	22 (100)
Malawi (MW)	1237 (37.6)	274 (28.5)	963 (71.5)	856 (34.3)	89 (11)	762 (89)
Rwanda (RW)	96 (2.9)	0	96 (100)	6 (0.2)	0	6 (100)
South Africa (ZA)	927 (28.2)	66 (7.1)	861 (92.9)	506 (20.4)	19 (3.6)	487 (96.4)
Tanzania (TZ)	36 (1.1)	2 (5.5)	34 (94.5)	174 (7)	55 (32.4)	119 (67.6)
Uganda (UG)	NA	NA	NA	1 (0.04)	0	1 (100)
Zambia (ZM)	962 (29.3)	42 (4.4)	920 (95.6)	875 (35.3)	61 (7)	814 (93)
Zimbabwe (ZW)	NA	NA	NA	26 (1)	2 (8.3)	24 (91.7)

*NA* not available

A total of 5769 sequences from both early and chronic heterosexual transmissions were analysed for X4 tropism. Overall, 385/3289 (11.7%; 95% CI − 1.8 to 13.9) of early infections were X4-tropic, versus 231/2480 (9.3%; 95% CI − 0.9 to 13.5) for chronic infections (p = 0.6446; X^2^ = 0.213). Country-wise, Malawi had the most X4 viruses in early heterosexual transmissions (28.5%; p = 0.0047; X^2^ = 8.000), and Tanzania had the most X4 viruses in chronic heterosexual transmissions (32.4%; p = 0.0001; X^2^ = 30.042), both of which were significant (Table 2).

### Co-receptor biotype prediction according to disease progression

Sequences from slow and rapid progressors from South Africa and Zambia were also available in significant numbers for analyses. There were no X4 tropic variants among the 145 early infection sequences from slow progressors. Among the 158 sequences from chronic slow progressors, 2/158 (1.3%; 95% CI 0–0) were X4 tropic, all from South Africa. The rapid progressors had a total of 169 early infections sequences none of which was X4; while 55/141 (39%) of the chronic sequences, were X4, from South Africa. A significantly higher proportion of sequences from chronic rapid progressors were of X4 tropic compared to those from chronic slow progressors (p = 0.001; X^2^ = 45.995) (Table 3).

**Table 3:** Co-receptor prediction using sequences from HIV-1 subtype C slow and rapid progressors during early and chronic infections

	Predicted biotype					
	Early			Chronic		
	Total (%)	X4 (%)	R5 (%)	Total (%)	X4 (%)	R5 (%)
Slow progressors						
Total sequences	145	0 (0)	145 (100)	158	2 (1.3)	156 (98.7)
South Africa (ZA)	63 (43.4)	0 (0)	63 (100)	158 (100)	2 (1.3)	156 (98.7)
Zambia (ZM)	82 (56.6)	0 (0)	82 (100)	NA	NA	NA
Rapid progressors						
Total sequences	169	0 (0)	169 (100)	141	55 (39)	86 (61)
South Africa (ZA)	97 (57.4)	0 (0)	97 (100)	141 (100)	55 (39)	86 (61)
Zambia (ZM)	72 (42.6	0 (0)	72 (100)	NA	NA	NA

95% confidence intervals for all the prevalences were zero

*NA* not available

### HIV-1 subtype C gp120 V3-loop diversity in Africa

**Fig. 2:**
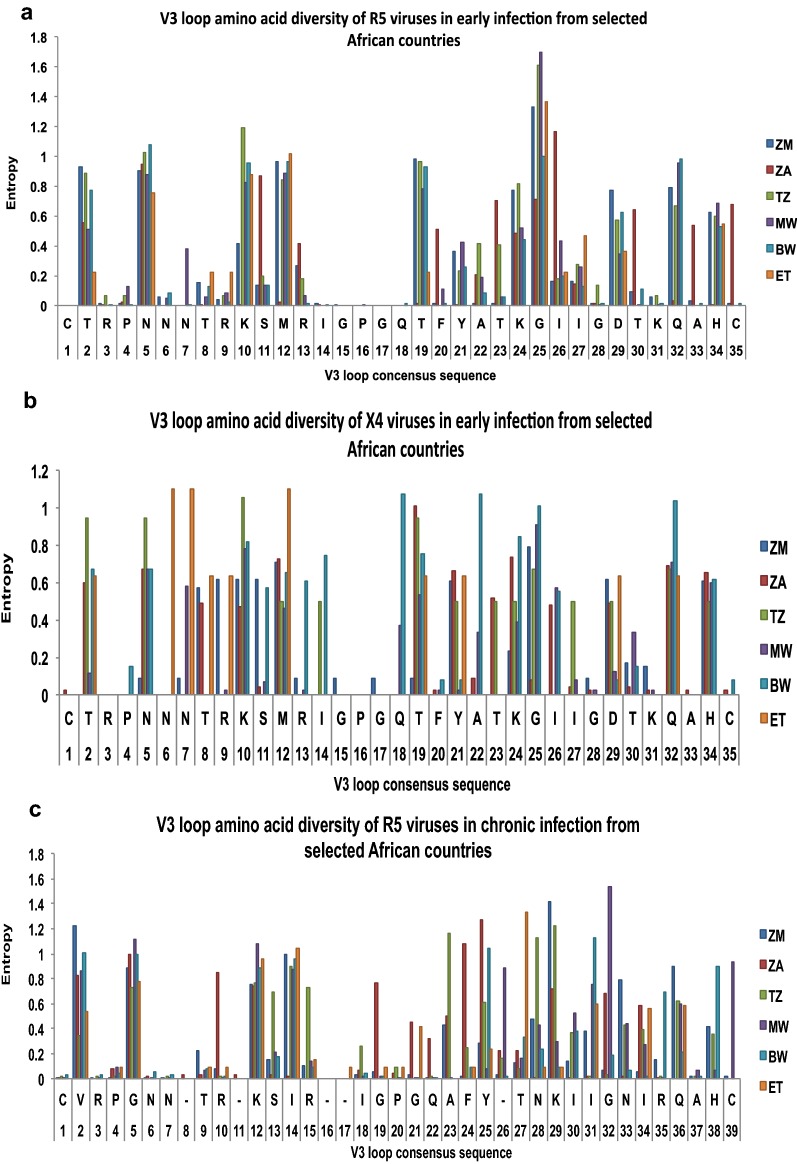

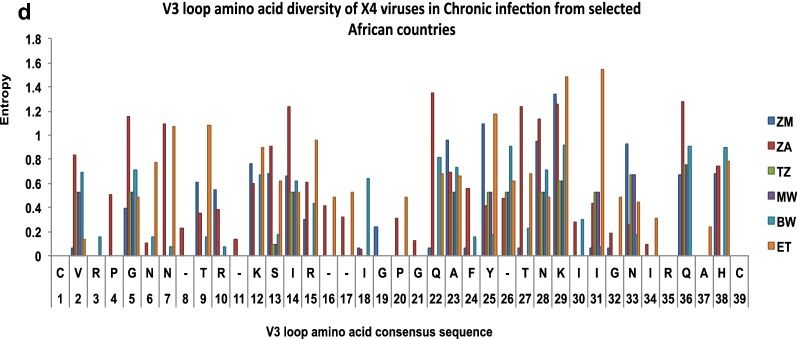
Inter-country variation within the V3 region of subtype HIV-1-C R5 and X4 viruses in early and chronic infection. **a**–**d** Are entropy plots for variations at each amino acid position for the different African countries

In order to perform V3-loop diversity analyses, sequences were available for seven countries, namely; Botswana, Ethiopia, Malawi, Tanzania, South Africa, Zambia, and Zimbabwe (Table 4). The least amino acid substitutions (measured by entropy) was observed in X4 and R5 sequences for both early and chronic infections from Botswana ranging from 0–1 and 0–1.25 respectively; while Ethiopia and Malawi had sequences with the highest entropy (range 0–1.915) in both early and chronic sequences of X4 and R5 variants. High levels of entropy were seen in positions 12, 25, 29 and 32 for both the R5 and X4 viruses (Fig. 2).

**Table 4:** HIV-1 subtype C V3 loop N-glycosylation site and V3 loop crown motif variation in African countries

Country	% of sequences that lost N-glycosylation site			
	Early		Chronic	
	R5	X4	R5	X4
Zambia (ZM)	4.5	17.2	5	30
South Africa (ZA)	0.1	18.4	0.7	6.2
Tanzania (TZ)	0	0	0	0
Malawi (MW)	1.1	0	1.3	22.7
Ethiopia (ET)	6	67	1.7	46.9
Botswana (BW)	4	0	2	3
Variation at the GPGQ Crown motif (%)				
Zambia (ZM)	0	→ RPGQ (1.8) → GPRQ (1.8)	→ GPGR (0.2) → RPRQ (0.4) → RPGQ (0.5) → GPEQ (0.07)	→ GPGR (1.25) → RPGQ (6.3)
South Africa (ZA)	→ GPGK (0.1) → GPGR (0.1)	0	→ GPGR (0.09) → GPGT (0.9) → GPGI (0.19)	→ GPGY (27.4) → GPGA (10.6) → GRGQ (7.1) → GPGT (4.4) → GRGQ (5.3) → GPGH (1.8) → GPRQ (1.8) → GPGL (0.9) → GQRQ (0.9)
Tanzania (TZ)	0	0	→ GPGH (0.3) → GLGQ (1.1) → GSGQ (0.6)	0
Malawi (MW)	0	→ GPGK (9.2) → GPGR (1.2)	→ GPGR (0.6) → GPGK (0.5) → GPGH (0.08) → EPGQ (0.04) → GRGH (0.04) → RPGQ (0.04)	→ GPGK (7.6) → RPGQ (0.8) → GSGQ (0.8)
Ethiopia (ET)	0	0	→ GPRT (1.7) → RPRQ (1.7)	→ GPGH (43.8) → GRGQ (18.8)
Botswana (BW)	→ GPGK (0.15)	→ GPGK (35.8) → GPGR (40.3)	→ GPGR (0.6) → RPGQ (0.3) → GQGQ (0.06) → GPGP (0.06) → GGGK (0.8)	→ GPGR (77) → GPGK (22.6)

**Fig. 3:**
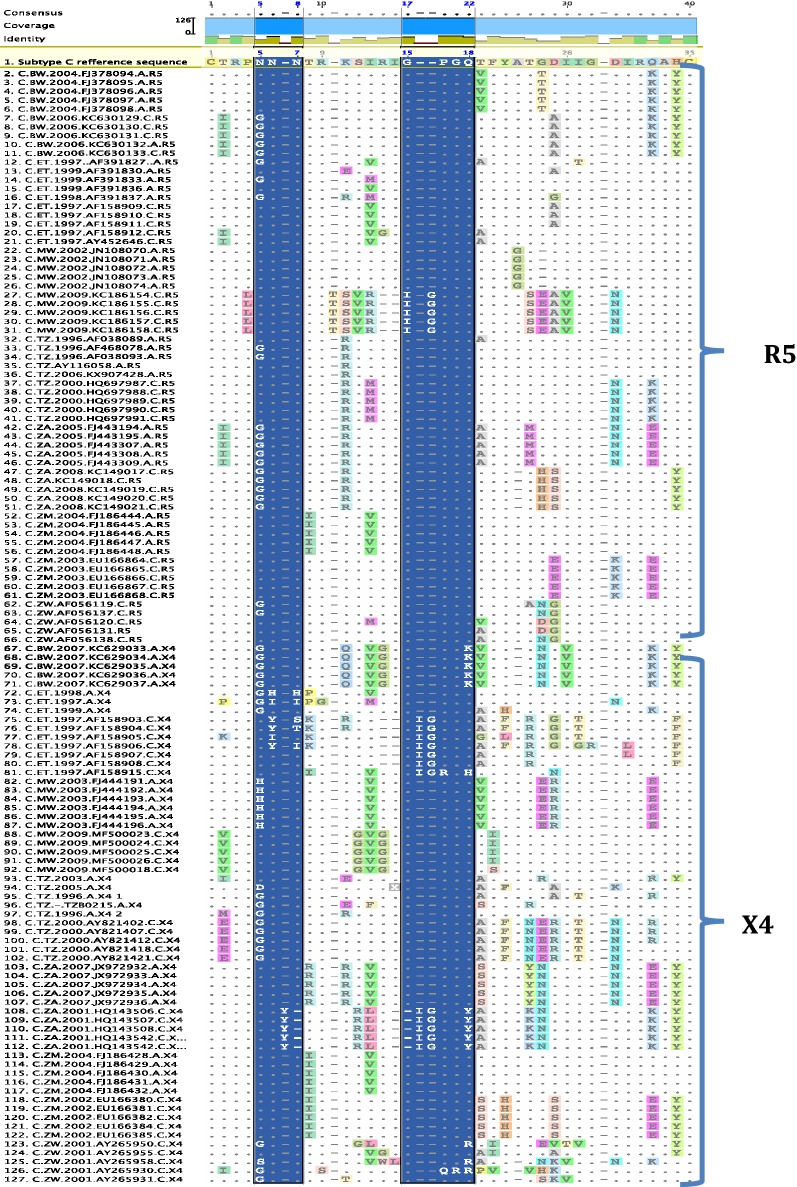
Amino acid alignment of 126 representative sequences of early and chronic R5 and X4 infections. Highlighted areas (blue) indicate the N-glycosylation site and the V3 loop crown motive

N-glycosylation sites play key roles in the interaction between the virus, CD4 molecule and the CCR5 and CXCR4 co-receptors; as well as aiding the virus to evade neutralization by host immune response. R5 and X4 viruses of early and chronic infections from Tanzania had a highly conserved N-glycosylation site while Botswana, Ethiopia, Malawi, South Africa, and Zambia had very few sequences that lost the N-glycosylation site (Table 4). On the contrary, sequences from Ethiopia showed the highest frequency of X4 viruses that had lost the N-glycosylation site for acute (67%) and chronic (46.9%) infections (Table 4, Fig. 3).

The crown motive (GPGQ) was very much conserved, except for Botswana for which about 77% of sequences from X4 viruses of early infections had Q → R (40.3%) and Q → K (35.8%) substitutions; while about 99% of sequences from chronic infections had Q → R (77%) and Q → K (22.6%) substitutions among the X4 viruses (Table 4). The amino acid lengths were shorter for early than chronic R5 or X4 viruses ranging between 34 and 35 for early and 34–38 for chronic infections. There was no particular trend in the V3-loop amino acid length across the countries (Table 5). The V3-loop net charge for both early and chronic X4 viruses were generally higher, ranging from 1 to 10, and lower in R5 viruses which ranged from 1 to 6 (Table 5).

**Table 5:** V3 loop amino acid diversity (amino acid length, deletions, insertions, net charge) of X4 and R5 viruses in early and chronic infections

Country	Early X4/R5				Chronic X4/R5			
	AA length	Deletion position	Insertion position	AA net positive charge	AA length	Deletion position	Insertion position	AA net positive charge
Zambia (ZM)	34–35/35	24/None	None/none	4–8/2–6	35/35	None/none	None/none	1–6/1–6
South Africa (ZA)	34–35/36	10, 23/22	None/9–10	4–8/2–6	34–37/34–35	22/22	7–8, 9–10, 13–14/none	4–10/2–6
Tanzania (TZ)	34–35/34–35	22, 24/22, 24	None/none	3–6/2–6	34–35/34–35	25/22, 23	None/none	4–6/1–6
Malawi (MW)	34–35/34–35	24/24	None/none	3–5/3–5	34–35/34–38	22, 25/24	None/13–14, 14–15	2–7/2–6
Ethiopia (ET)	35/35	None/none	None/none	4–6/2–5	35–37/35	None/none	12–13/none	3–8/0–6
Botswana (BW)	35/35	None/none	None/none	3–6/1–6	34–35/34–37	22/22	None/13–14	2–6/1–6

**Fig. 4:**
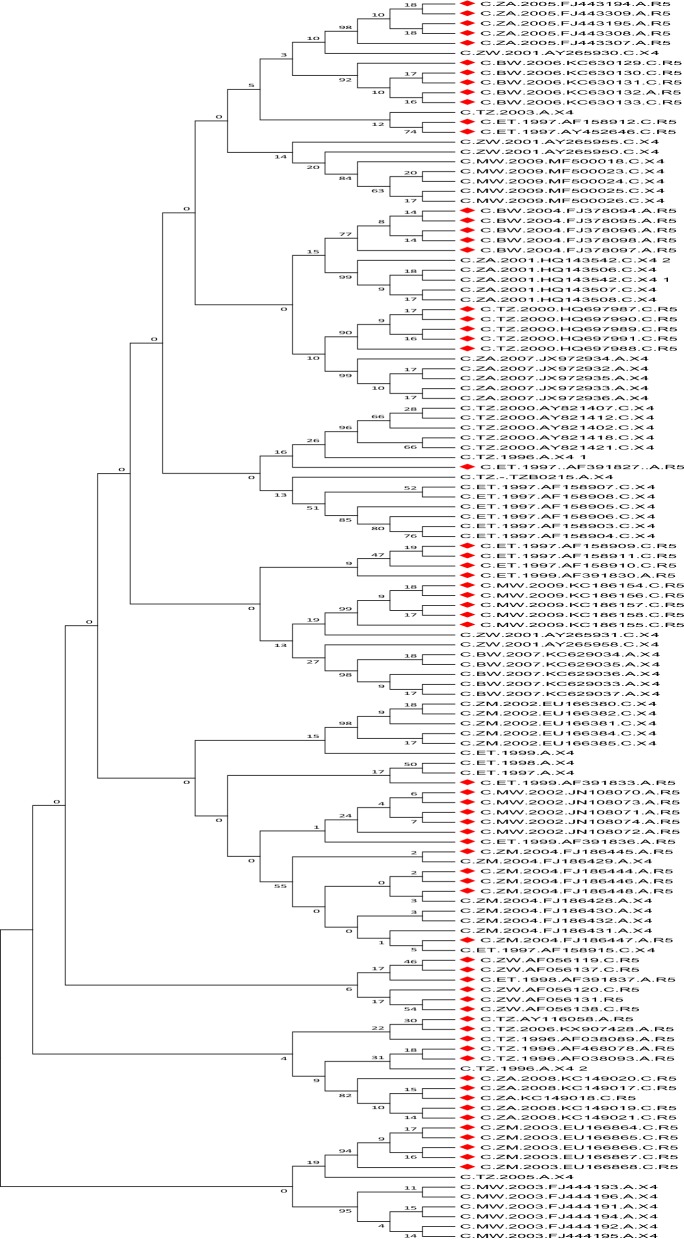
Neigbour joining phylogenetic tree of 126 representative sequences of early and chronic R5 and X4 infections from 6 African countries. Majority of these sequences cluster based on predicted biotypes

As expected, a higher level of gp120 V3-loop amino acid variation was observed in X4 tropic viruses from chronic than early infections, with South Africa, Ethiopia and Zimbabwe showing the highest levels of V3-loop diversity (Fig. 2). All sequences were phylogenetically confirmed as HIV-1 subtype C. More than 92% of the sequences clustered according to their tropism. Only two R5 sequences (3%) and eight X4 sequences (13%) did not cluster according to their tropism based on the selection of sequences used for the phylogenetic analysis (Fig. 4).

### Use of Maraviroc in Africa

A scoping review of literature on the active use of Maraviroc revealed Tanzania and Zambia as the only countries in Africa that include Maraviroc as a component of salvage therapy in their national ART guidelines, following an HIV tropism test. Although the Southern African HIV Clinicians Society recommends the use of Maraviroc in salvage therapy, this has not been incorporated in the treatment guidelines by the Health Ministries of Southern African Countries. In addition, Maraviroc is registered in South Africa and Uganda. Table 6 shows details for countries on the registration and or recommendation on the use of Maraviroc.

**Table 6:** Current registration and recommended use of Maraviroc in African countries

African countries	Year of registration (Registering authority)	Recommendation in treatment guidelines/remarks	URL links
South Africa	2013 (Medicines Control Council)	Not included in the national treatment guidelines, but the Southern African HIV Clinicians Society recommends its use as salvage therapy	https://www.sahpra.org.za/documents/149a634812.71_Notification_of_Registration_Mar13_v1.pdf Accessed 20 Jan 2020 https://sahivsoc.org/Files/2014%20Adult%20ART%20Guideline%20(Dec%202014).pdf Accessed 14 Jan 2020
Tanzania	Information on registration or of the registering authority is not available	Recommended in the national treatment guidelines as a component in third line treatment by the Ministry of Health, Community Development, Gender, Elderly, and Children	https://www.google.com/url?sa=t&rct=j&q=&esrc=s&source=web&cd=1&ved=2ahUKEwj3rtrNsIXnAhV2QkEAHSBnDuoQFjAAegQIARAC&url=http%3A%2F%2Fwww.nacp.go.tz%2Fsite%2Fdownload%2FNATIONAL_DECEMBER_2017.pdf&usg=AOvVaw0XAyt4IPPIA8OE_-qSR9no Accessed 14 Jan 2020
Uganda	2008 (National drug register of Uganda Human Medicines)	Currently not recommended in the national treatment guidelines	https://www.nda.or.ug/Linked%20publications/NATIONAL%20DRUG%20REGISTER%20HUMAN%20JANUARY%202020.pdf Accessed 14 Jan 2020
Zambia	Information on registration or the registering authority is not available	Recommended in the national treatment guidelines as a component in third line treatment by the Ministry of Health	http://www.hivst.org/files1/Final-Zambia-Consolidated-Guidelines_2018-Print.pdf Accessed 14 Jan 2020

In these four countries, a specialist committee recommends the use of maraviroc based on HIV drug resistance genotyping data for virologic failure, and HIV tropism test

## Discussion

Maraviroc is a CCR5 antagonist, that prevents HIV from utilizing the CCR5 co-receptor to enter target cells. During the acute phase of infection, HIV strains irrespective of genotype, utilize CCR5 as the main co-receptor. However, as disease progresses, CXCR4 utilizing viruses emerge in about 50% of infected individual [41–43]. By and large, in Africa Maraviroc is prescribed mostly for patients who have failed first and second line regimens, which are comprised of nucleoside reverse transcriptase inhibitors, non-nucleoside reverse transcriptase inhibitors, and protease inhibitors [44, 45]. Since, HIV-1-C drives the epidemic in Southern Africa and accounts for about 46% of infections worldwide [24], the current investigation was aimed at determining the distribution of R5 and X4 viruses in early versus chronic infections in HIV-1-C acquired through MTCT and heterosexual routes in Africa.

Using predicted biotypes of about 14,641 gp120 V3-loop sequences, filtered from the Los Alamos HIV database, our analyses showed that X4 variants are present in significantly similar proportions in early and early chronic (< 1 year post-infection) HIV-1-C infected individuals. However, in late chronic infections (5 years post infection), X4 variants increase 3–5 folds. A study by [46], studying paired RNA and proviral DNA from HIV-1-B antiretroviral naive patients with acute and chronic infections, showed that more than 90% of viruses in acute infections from plasma and peripheral blood mononuclear cells were R5, while patients with chronic infections had a significantly higher prevalence of X4 viruses than in patients with acute infection. Another study [47], evaluating 200 patients for co-receptor tropism, using an ultra-deep sequencing approach, also showed that both X4 and R5 viruses co-exists during acute infections, but with R5 viruses as the highly predominant variant. This shift from R5 to X4 viruses was also observed in the current study.

Several hypothesis have been advanced to explain the predominance of R5 viruses in early infection and X4 viruses in chronic infections: that X4 and R5 viruses are transmitted at the same time but X4 viruses are suppressed by the prevailing strong immune response at the time of infection and proliferate later in infection when the immune surveillance is weak [48–50]; that X4 viruses emerge from R5 viruses at the beginning of infection [51, 52]; and thirdly, X4 and R5 viruses have different target cell types, with more target cell types for X4 viruses (T-cells) increasing in abundance as infection becomes chronic [53, 54]. A closer look at the sequences in the current study showed that overall, the prevalence of X4 viruses in early infections and early chronic (between 6 months and 1 year post-infection) are similar. However, there is a dramatic rise in the frequency of X4 viruses when sequences from patients with more than 5 years of infection were considered. The inference is that within 4 years of infection with HIV-1-C, the proportion of X4 viruses becomes significantly higher ranging from 24 to 43%. In fact in a recent study, we reported a high frequency (43%) of X4 viruses identified by ultra-deep sequencing from a cohort of HIV-1-C chronically infected individuals from northern South Africa [34]. Overall in the current study, the prevalence of X4 viruses was not significantly different in early and chronic infections among individuals who were vertical infected, and was also similar among individuals who acquired infection heterosexually. It also appears the route of infection does not influence coreceptor tropism in early or chronic infections. We did not find X4 viruses in early infections from both slow and rapid progressors; but there was significantly more X4 viruses in chronic rapid progressors than in chronic slow progressors.

The findings from the current study should be examined in the context of several limitations. Firstly, there was a dearth of sequence data from longitudinal cohorts in Africa to assess evolution of co-receptor usage over time. Nevertheless, in seeking correlates of co-receptor switch between CCR5 and CXCR4 [55], reported no significant difference in co-receptor ‘switching’ over time among patients who were initially infected exclusively with R5 or X4 viruses, when age, viral load, and gender was considered. In the same vein [56], showed that viruses are either R5 or X4 but not dual tropic, and that dual tropism is due to mixture of both phenotypes. Secondly, data meeting the selection criteria for known acute infections and routes of transmission on HIV-1-C infections were available for only 15 African countries, with four countries (Botswana, Malawi, South Africa and Zambia) providing a highly disproportionate number of sequences. This limits the scope of applicability of the findings at least in Southern Africa where HIV-1-C predominantly drives the epidemic. Thirdly, due to the degree of false prediction of co-receptor usage by bioinformatic tools such as Geno2Pheno and position-specific scoring matrices, opinion on the clinical usefulness of predicted coreceptor usage varies [57–59]; so predictions will need to be seen in the context of other clinical parameters. Nevertheless, with the observation that X4 viruses exist in an appreciable proportion in chronic infections and that Maraviroc usage as salvage therapy might lead to resistance due to pre-selection of X4 strains [60], it is intriguing why Maraviroc should be reserved for management at the late stage of infection even in those few African countries in which it is recommended for salvage therapy. Nevertheless, new changes in treatment regimens are being introduced. In an effort to reduce drug resistance to non-nucleoside reverse transcriptase inhibitors (NNRTI), many low and middle income countries, and also high income countries are replacing NNRTI with dolutegravir, an integrase strand transfer inhibitors (INSTI), in first and second line treatment regimens [61, 62]. For example, recently in November 2019, South Africa switched from a fixed dose combination of standard tenofovir–lamivudine–nevirapine to a fixed dose combination of tenofovir–lamivudine–dolutegravir. This move may potentially delay, across the board, the use of Maraviroc in patient management.

## Conclusion

Our data show that the use of Maraviroc is very limited in Africa, and confirms that for an improved utility of Maraviroc as salvage therapy among HIV-1-C patients in Africa, preliminary virus co-receptor determination is required. Alternatively, Maraviroc may be included as first line therapy in combination with nucleoside analogues; although this may not be beneficial if prevention of mother-to-child transmission is a desirable outcome, since there is no evidence of Maraviroc’s efficacy in the prevention of HIV mother-to-child transmission. Finally, research in CXCR4 antagonists is encouraged as universal access to treatment gains steam across Africa.

## Data Availability

Sequences used for this analysis were obtained from the Los Alamos HIV Sequence Database. The extracted dataset is available upon request.

## Abbreviations

cART: Combination antiretroviral therapy
CCR5: C-C chemokine receptor type 5
CXCR4: C-X-C chemokine receptor type 4
HIV-1-C: HIV subtype C
R5: CCR5 utilizing viruses
MTCT: Mother to child transmission
X4: CXC4 utilizing viruses
UNIAIDS: Joint United Nations Programme on HIV/AIDS
